# Technique, pitfalls, quality, radiation dose and findings of dynamic 4-dimensional computed tomography for airway imaging in infants and children

**DOI:** 10.1007/s00247-018-04338-5

**Published:** 2019-01-25

**Authors:** Savvas Andronikou, Mark Chopra, Simon Langton-Hewer, Pia Maier, Jon Green, Emma Norbury, Sarah Price, Mary Smail

**Affiliations:** 1 Department of Paediatric Radiology, Bristol Royal Hospital for Children, University Hospitals Bristol NHS Foundation Trust, Bristol, UK; 2 Department of Paediatric Radiology, University of Bristol, Bristol, UK; 3 Department of Pediatric Radiology, Section of Pulmonary Imaging, Children’s Hospital of Philadelphia, 3NW 39, 3401 Civic Center Blvd., Philadelphia, PA 19104 USA; 4 Department of Paediatric Pulmonology, Bristol Royal Hospital for Children, Bristol, UK; 5 Radiation Science Services, Medical Physics & Bioengineering, University Hospitals Bristol NHS Foundation Trust, Bristol, UK

**Keywords:** Airways, Bronchus, Child, Computed tomography, Trachea, Tracheobronchomalacia, Radiation dose

## Abstract

This retrospective review of 33 children’s dynamic 4-dimensional (4-D) computed tomography (CT) images of the airways, performed using volume scanning on a 320-detector array without anaesthesia (free-breathing) and 1.4-s continuous scanning, was undertaken to report technique, pitfalls, quality, radiation doses and findings. Tracheobronchomalacia (airway diameter collapse >28%) was recorded. Age-matched routine chest CT scans and bronchograms acted as benchmarks for comparing effective dose. Pitfalls included failure to administer intravenous contrast, pull back endotracheal tubes and/or remove nasogastric tubes. Twenty-two studies (67%) were diagnostic. Motion artefact was present in 16 (48%). Mean effective dose: dynamic 4-D CT 1.0 mSv; routine CT chest, 1.0 mSv, and bronchograms, 1.4 mSv. Dynamic 4-D CT showed tracheobronchomalacia in 20 patients (61%) and cardiovascular abnormalities in 12 (36%). Fourteen children (70%) with tracheobronchomalacia were managed successfully by optimising conservative management, 5 (25%) underwent surgical interventions and 1 (5%) died from the presenting disorder.

**Electronic supplementary material:**

The online version of this article (10.1007/s00247-018-04338-5) contains supplementary material, which is available to authorized users.

## Introduction

Some extended detector row multi-detector CT scanners allow for volumetric imaging of the entire airway during free breathing without having to move the patient through the gantry. This avoids misregistration of the airway position due to normal craniocaudal movement of the airway during respiration and allows 3-D reconstruction and viewing of cine-loops during breathing [1]. This technique is ideal for evaluating tracheobronchomalacia in children.

Tracheobronchomalacia is defined as excessive collapsibility of the airway, which is either idiopathic or secondary to extrinsic compression [2, 3]. Bronchoscopic diagnosis is subjective and defined as proportional airway collapse of more than 50% of the lumen, compared to the normal airway, under self-ventilation [4, 5]. For a classical imaging diagnosis, a proportional collapsibility of the airway of greater than 50% must be demonstrated [2]. In meeting this definition, tracheograms, bronchograms and fluoroscopy cannot quantify the airway cross-sectional area but instead quantify anteroposterior diameter [6]. Dynamic four-dimensional (4-D) computed tomography (CT) provides images for subjective evaluation and more objective measurement of airway collapse, noninvasively, through volume imaging of the whole airway during free breathing, without moving the patient through the gantry and by providing information on airway dynamics in a cine-loop format [1, 3]. For free-breathing dynamic airway assessment using dynamic 4-D CT, tracheobronchomalacia has been defined as airway diameter reduction of more than 28% [1, 7].

Limited availability of 320-detector row CT scanners with volume scanning capabilities and the perception that dynamic 4-D CT imparts a higher radiation dose than bronchography have restricted its use [8]. We aim to present our early experiences with dynamic 4-D CT in children to assist those wishing to start such a program.

### Description

We describe our experience through a retrospective review of dynamic 4-D CT scans of the airways performed in children younger than 18 years at one children’s hospital over a 22-month period (2015 to 2017).

Our standard technique uses a 320-detector array Aquilion One Vision Edition (Toshiba Medical Systems Corporation, Otawara, Japan) with 16-cm Z-axis coverage and maximum rotation speed of 0.275 s. Scans are performed without anaesthesia, under free breathing when possible and with physical restraint of the patient as necessary. In the setting of an intubated and paralysed child, positive pressure ventilation settings are set as low as safely possible or turned off. Maximum respiratory rate for intubated patients is set to 40 breaths/min corresponding to one respiratory cycle during dynamic scanning. We follow a protocol modified from Greenberg and Dyamenahalli [7]:

$$ tube\kern0.17em current(mA)=\frac{\left[\left( body\kern0.17em weight(kg)\times 1.5\right)+5\right]}{0.35} $$

We apply 80-kVp continuous scanning for 1.4 s (4 cycles at 350 ms/rotation) (or 5 cycles at 275 ms/rotation) and reconstruction of 8–10 phases [7]. We customise the scan range from the thoracic inlet to just beyond the major bronchi (well above the diaphragm) using the scanogram. Three-dimensional (3-D) volume-rendered reconstructions and minimum intensity projections are created for viewing in cine mode (Supplementary material 1) alongside axial source images.


Supplementary material 1Transparent 3-D volume-rendered reconstruction of a dynamic 4-D CT scan of the airways in a 4-year-old girl with suspected but not proven tracheobronchomalacia, viewed anteriorly in cine mode, demonstrates normal craniocaudal movement of the major airways during free breathing, and no dynamic or fixed narrowing. (MP4 1,593 kb)


### Evaluation

Two subspecialist paediatric radiologists (one with more than 20 years of experience (SA) and one with 2 years of experience (MC)) evaluated dynamic 4-D CT scans retrospectively, quantifying pitfalls encountered, assessing quality and documenting artefacts. Estimated effective dose was determined by two medical physicists. Fifteen age-matched routine CT chest scans for other indications acted as controls. Eight bronchograms of age-matched children were used for dose benchmarking.

Pitfalls recorded included: (a) scanning without intravenous contrast, (b) scanning with an endotracheal tube in situ and (c) scanning with a nasogastric tube in situ. Quality was assessed as good, acceptable or poor and nondiagnostic according to criteria summarized in Table 1. Reviewers also documented the presence of air in the oesophagus, hampering 3-D reconstructions. Effective dose was calculated from the dose-length product using the equation effective dose=k x dose-length product. The conversion factors (k), provided in Deak et al. [9], are given as a function of kV (80 kV), body region (chest) and patient age for International Commission on Radiological Protection Publication 103 recommendations [10]. The same was done for calculating effective dose for routine chest CT on the same equipment. Effective dose was calculated for the eight consecutive paediatric bronchograms (biplane fluoroscopy; Siemens Artis zee, Forchheim, Germany) by entering patient x-ray exposure parameters from the study report, and dose area product into a Monte Carlo-based computational program (PCXMC; Version 2.0.1.3) (STUK, Helsinki, Finland).

**Table 1:** Quality categories for dynamic 4-D CT

Category	Criteria
Good - diagnostic	Successfully performed without motion or density artefact, i.e. all of these criteria: a) Included all the relevant anatomy/pathology. b) Clearly included an inspiratory and an expiratory phase (as determined by a clear motion of the airway in a craniocaudal direction and with clear change in the caliber of the airway in the axial plane). c) Not degraded by motion or density artefact.
Acceptable - diagnostic	Achieved with motion or density artefact but remained interpretable, i.e. all of these criteria: a) Included all the relevant anatomy/pathology. b) Included an inspiratory and an expiratory phase (as determined by a clear motion of the airway in a craniocaudal direction and with clear change in the caliber of the airway in the axial plane). c) Degraded by motion or density artefact that did not prevent evaluation and measurement of the airway collapsibility in the areas of interest.
Poor and nondiagnostic	Achieved incompletely, with motion or density artefact that made it uninterpretable, i.e. any of these criteria: a) Did not include all the relevant anatomy/pathology. b) Did not include an inspiratory and an expiratory phase (as determined by a clear motion of the airway in a craniocaudal direction and with clear change in the caliber of the airway in the axial plane). c) Significant artefact caused misregistration of structures during respiratory phases on reconstructions. d) Significant artefact caused obscuration of relevant pathology/anatomy. e) Significant artefact precluded evaluation and measurement of the airway collapsibility in the areas of interest.

Airway stenosis was determined during all phases of respiration (Supplementary material 2) by subjective agreement of the two paediatric radiologists. Classical tracheobronchomalacia was objectively determined by measuring airway diameter reduction of more than 28% in any plane from 3-D reconstructions visualised through the respiratory cycle (Supplementary material 3) based on revised criteria of Greenberg [1] and Greenberg and Dyamenahalli [7] for free breathing. In the scenario of preexisting airway stenosis on inspiration, an additional criterion for tracheobronchomalacia was collapsibility >28% on expiration, calculated proportionally against normal airway just proximal to the stenosis.


Supplementary material 2Transparent 3-D volume-rendered reconstruction of a dynamic 4-D CT scan of the airways in a 5-month-old, viewed in cine mode from an anterior vantage, demonstrates a severe stenosis of the left main bronchus that shows very little change between inspiration and expiration. (MP4 966 kb)
Supplementary material 3Transparent 3-D volume-rendered reconstruction of a dynamic 4-D CT scan of the airways in a 6-year-old girl, viewed in cine mode obliquely from the left side, demonstrates idiopathic bronchomalacia involving the bronchus intermedius and right upper lobe bronchus, i.e. there is reduction of more than 28% of the bronchial dimensions during free-breathing expiration. (MP4 1,522 kb)


No ethics clearance was required for this retrospective review per institutional guidelines and patient confidentiality was maintained.

### Findings

Thirty-three dynamic 4-D CT scans were performed over 22 months (19 [58%] boys; 14 [42%] girls; age range: 0.13 years–6.4 years; mean age: 1 year and 3 months) (Table 2).

**Table 2:** Summary of dose parameters and effective dose calculations of the 33 dynamic 4-D CT scans versus 15 routine CT scans in age-matched controls and the 8 most recent bronchograms in children

	Dynamic 4-D CT (*n*=33)	Routine CT chest (*n*=15)	Bronchograms (*n*=8)
Age range	0.1–6.4 years	0.1–6.8 years	0.1–0.6 years
Age mean	1.3 years	1.0 years	0.4 years
kV	80	80	66–71 average kV: 67.8
mAs	2–35 average mAs: 15.9	24–71 average mAs: 14.2	^a^95–401 average mAs: 211.9
CT dose index_Vol_ range	0.7–3.7 mGy	0.6–1.8 mGy	n/a
CT dose index_Vol_	2.3 mGy	1.3 mGy	n/a
Dose-length product/dose area product range	4.3–39.7 mGycm	5.9–40.1 mGycm	8.6–151.1 μGym^2^
Dose-length product/dose area product mean	17.9 mGycm	24.2 mGycm	53.0 μGym^2^
Effective dose range	0.3–2.7 mSv	0.5–1.9 mSv	^b^0.3–3.5 mSv
Effective dose mean	1.0 mSv	1.0 mSv	*1.4 mSv

^a^Value is average mA

^b^Using PCXMC Monte Carlo Dose Simulation

*4-D* four-dimensional, *CT* computed tomography, *vol* volume

#### Indications (Fig. 1)

All patients were referred for evaluation of tracheobronchomalacia – 14 (42%) because of underlying cardiovascular disease, 4 (12%) with conditions known to predispose to tracheobronchomalacia (a history of oesophageal atresia and tracheaoesophageal fistula, prolonged intubation or complications with general anesthesia) and 15 (45%) were referred because of persistent respiratory systems without a known predisposition for tracheobronchomalacia.

**Fig. 1:**
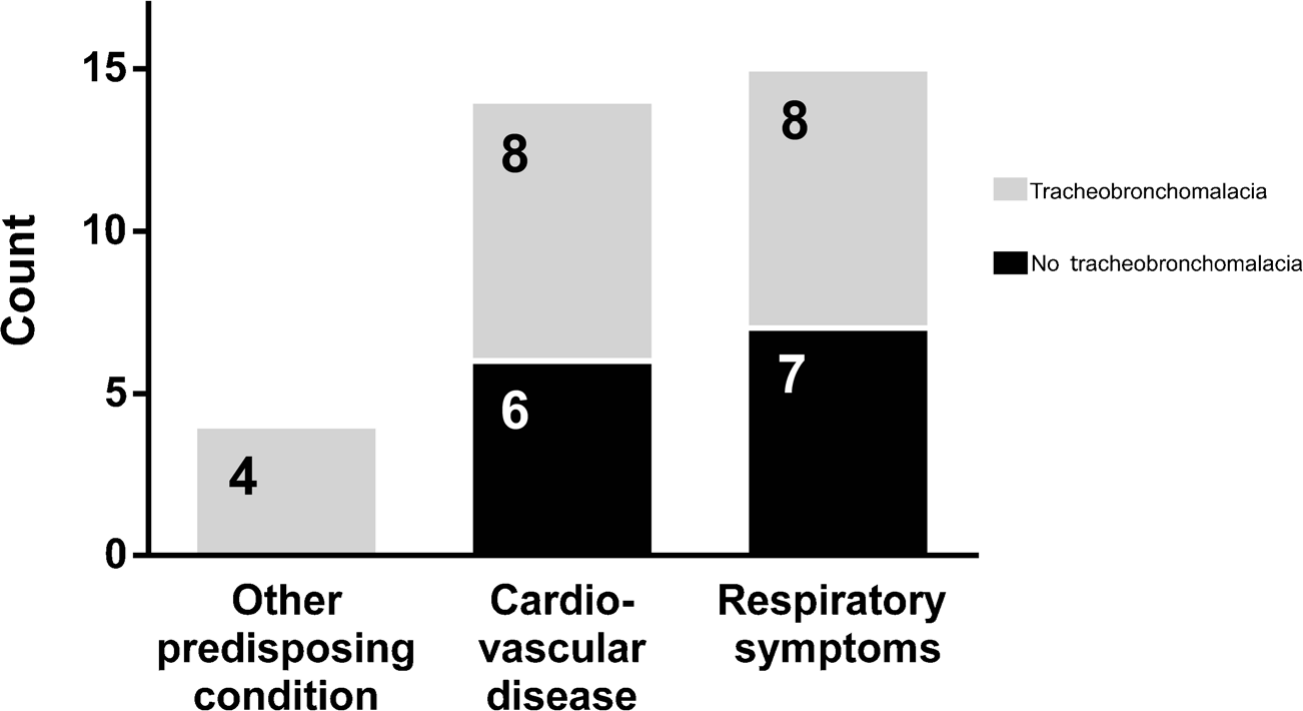
Bar graph summarises the referral indications for the 33 patients referred to dynamic 4-D CT as well as the number of patients within each group who were demonstrated to have tracheobronchomalacia. Other predisposing condition refers to two patients with a history of tracheo-oesophageal fistula, one with prolonged intubation and one with complications during general anaesthesia

#### Pitfalls

**Fig. 2:**
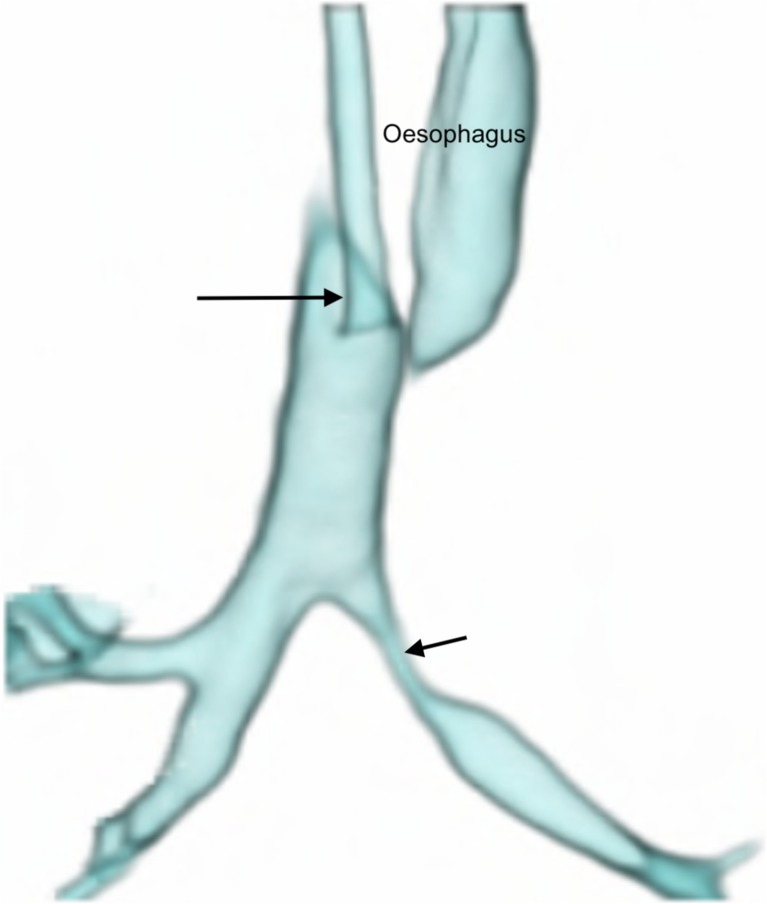
Anterior view of a volume-rendered reconstruction from a dynamic 4-D CT study of the airways in a 1-month-old girl with stenosis of the left main bronchus (*short arrow*). The distal two-thirds of the trachea could be evaluated in this instance because the endotracheal tube was withdrawn to lie with its tip in the upper third of the trachea (*long arrow*) using the planning scannogram (not shown). Oesophageal air is present and appears on the airway 3-D reconstruction setting. Despite efforts to cut this manually from all four or five phases of the scan, it is often not possible to achieve (as in this case) without encroaching on parts of the airway, due to normal craniocaudal and anteroposterior movement of the airway within the imaged volume, during breathing

We failed to administer intravenous contrast in 11 patients (33%), precluding identification of vascular causes; intravenous contrast is now routine. We scanned 6 patients (18%) with indwelling endotracheal tubes, but after the first nondiagnostic study due to tube artefact, we withdrew all endotracheal tubes into the upper third of the trachea (Fig. 2). We performed 15 procedures (45%) with indwelling nasogastric tubes, which we now remove routinely.

#### Quality

**Fig. 3:**
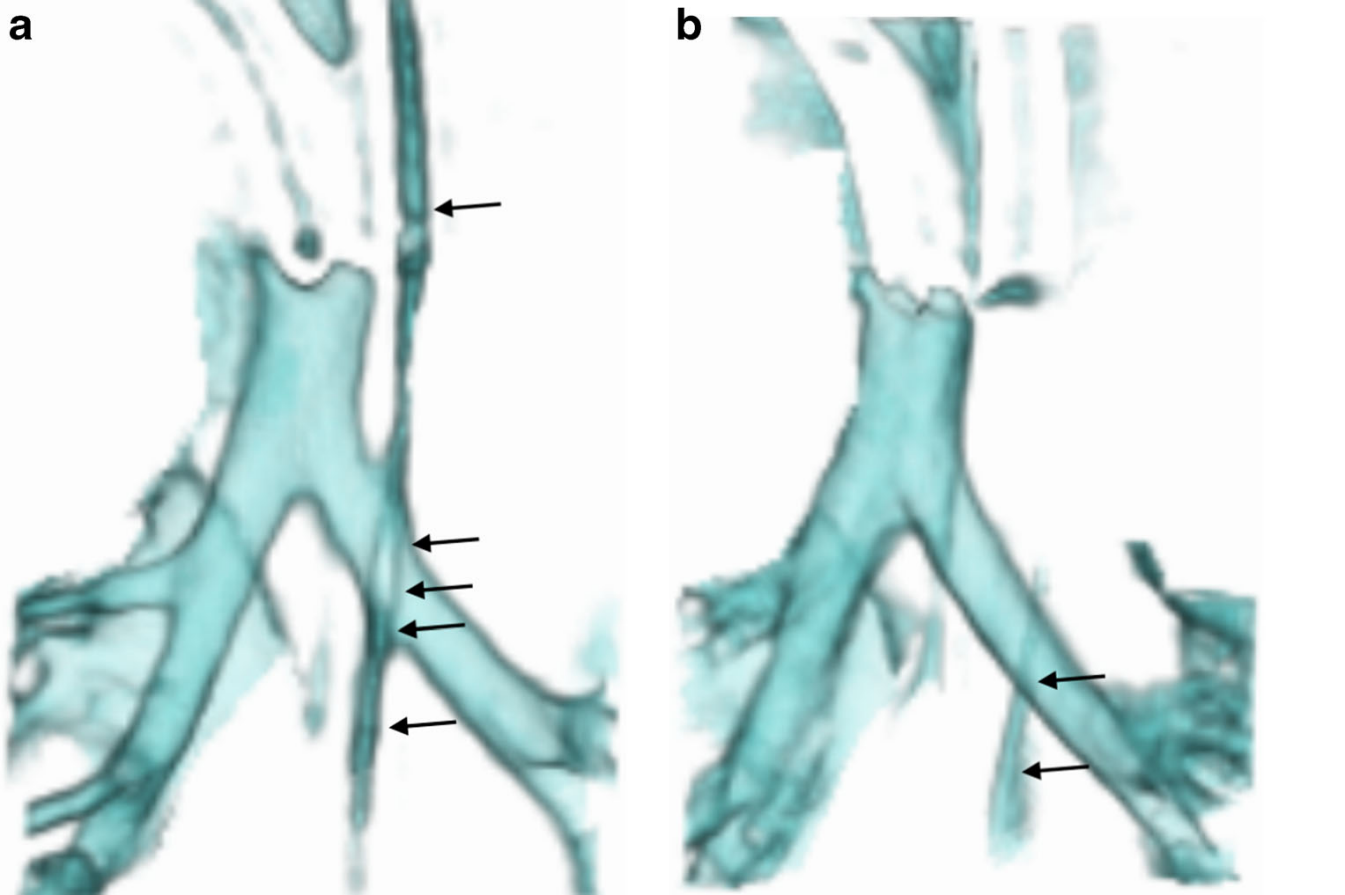
Anterior views of 3-D volume-rendered reconstructions of two of the five phases of a dynamic 4-D CT study of the airways in an intubated and ventilated 1-month-old boy with suspected tracheobronchomalacia. **a**, **b** Images from two different phases of ventilation demonstrate the nasogastric tube (*arrows*) crossing the left main bronchus and distorting the outline of the airway during inspiration (**a**) but not during expiration (**b**), which could affect assessment of collapsibility of the airway

**Fig. 4:**
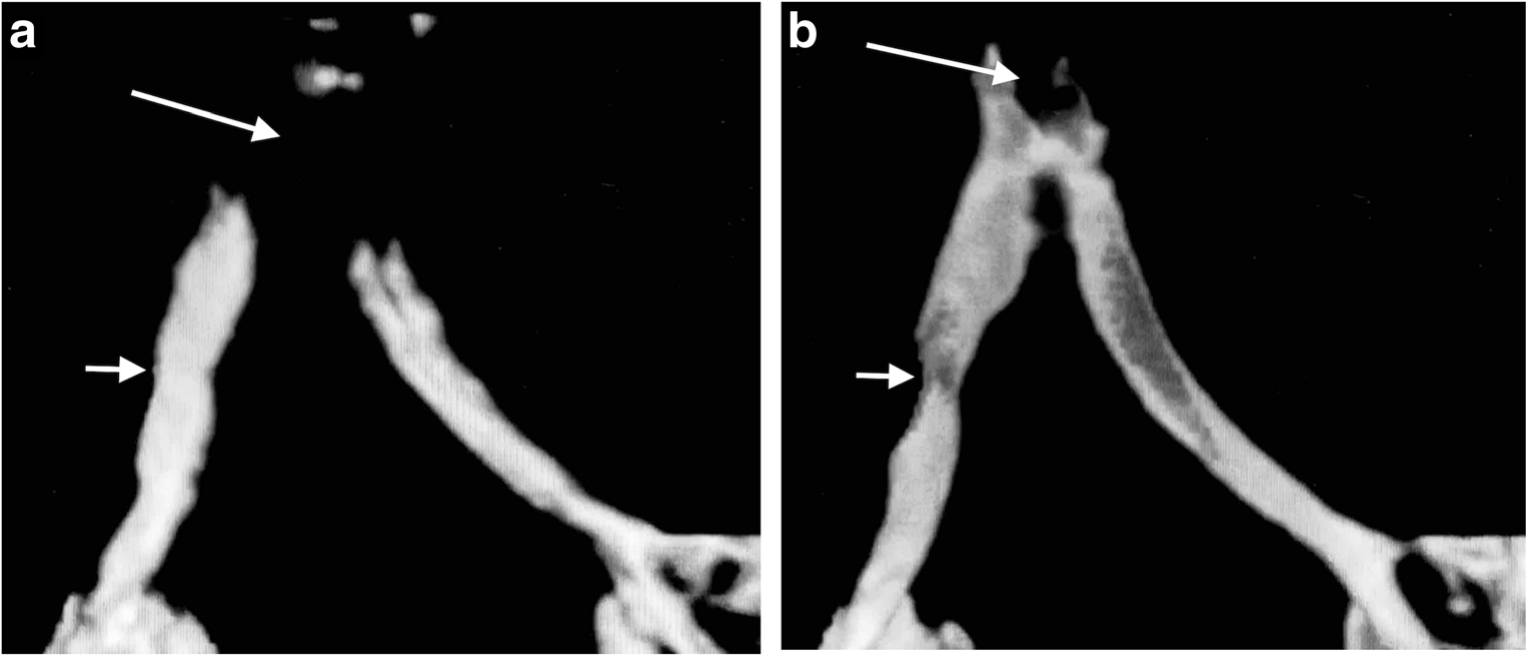
Frontal views of a dynamic 4-D CT performed in a 6-month-old girl with bronchomalacia involving the bronchus intermedius. **a, b** Frontal views of the volume-rendered reconstructions of two out of four phases of a dynamic 4-D CT study of the airways demonstrate an artefact arising from the endotracheal tube tip positioned at the carina (*long arrows*), which precludes evaluation of the trachea. The tube position changes from inspiratory phase (**a**) to the expiratory phase (**b**), which demonstrates the bronchomalacia involving the bronchus intermedius (*short arrows*). Despite demonstrating the bronchomalacia, this study was recorded as nondiagnostic because it failed to evaluate the trachea

**Fig. 5:**
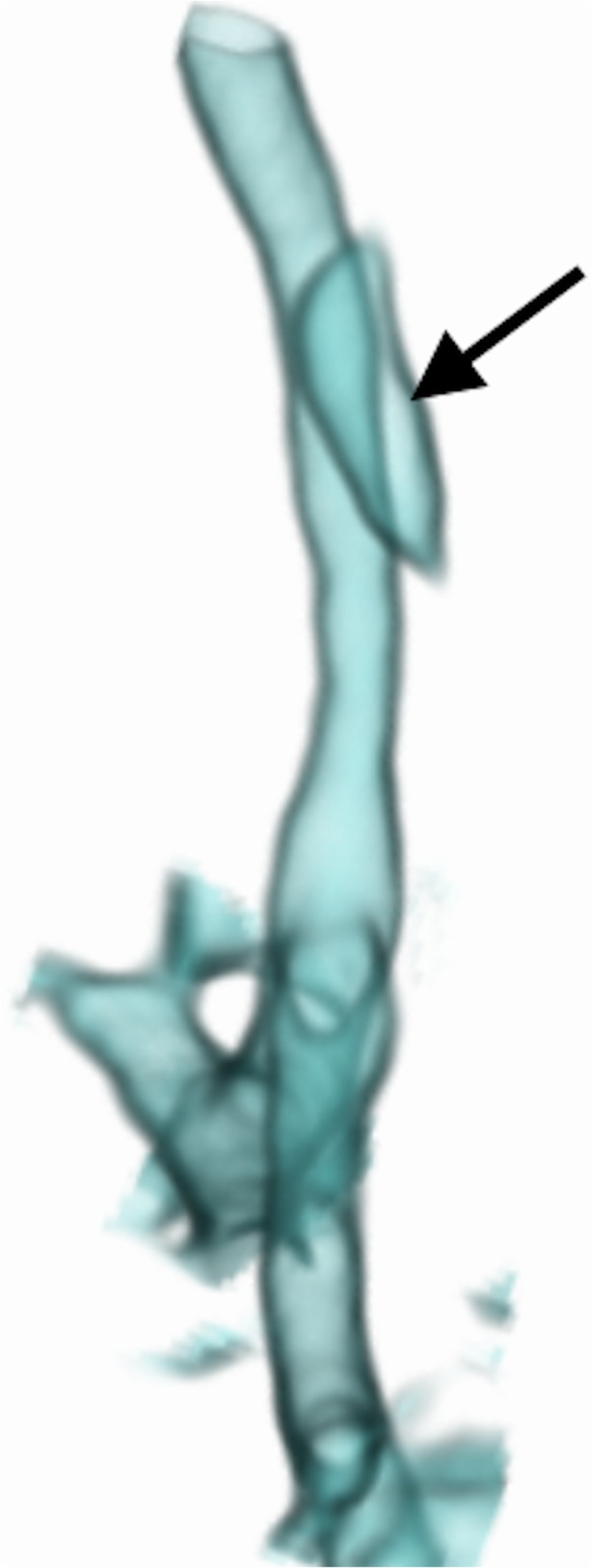
Lateral view of the volume-rendered reconstruction of a dynamic 4-D CT study of the airways in a 4-month-old boy with suspected tracheobronchomalacia, demonstrating the problem posed by gas in the oesophagus (*arrow*) overlapping the trachea. It could not be cut without compromising the airway over the range of phases, due to normal airway movement within the scanned volume


Eleven (33%) studies were poor/nondiagnostic while 22 (67%) were diagnostic -- 16 good (48%) and 6 acceptable (18%). Artefact was seen in 18 cases (54%) -- motion in 16 (48%) (Supplementary material 4); nasogastric tube in 6 (18%) (Supplementary material 4) (Fig. 3), endotracheal tube in 1 (3%) (Fig. 4) and metal in 1 (3%). Oesophageal gas was present in 10 cases (30%), affecting reconstructions (Figs. 2 and 5).

Supplementary material 4Transparent 3-D volume-rendered reconstruction in a dynamic 4-D CT scan of the airways in a 1-month-old boy, viewed in cine mode from an anterior viewpoint, demonstrates significant movement of the airways caused by patient motion and artefact caused by the nasogastric tube overlying the left main bronchus making the study nondiagnostic. (MP4 3,218 kb)


#### Radiation dose

**Fig. 6:**
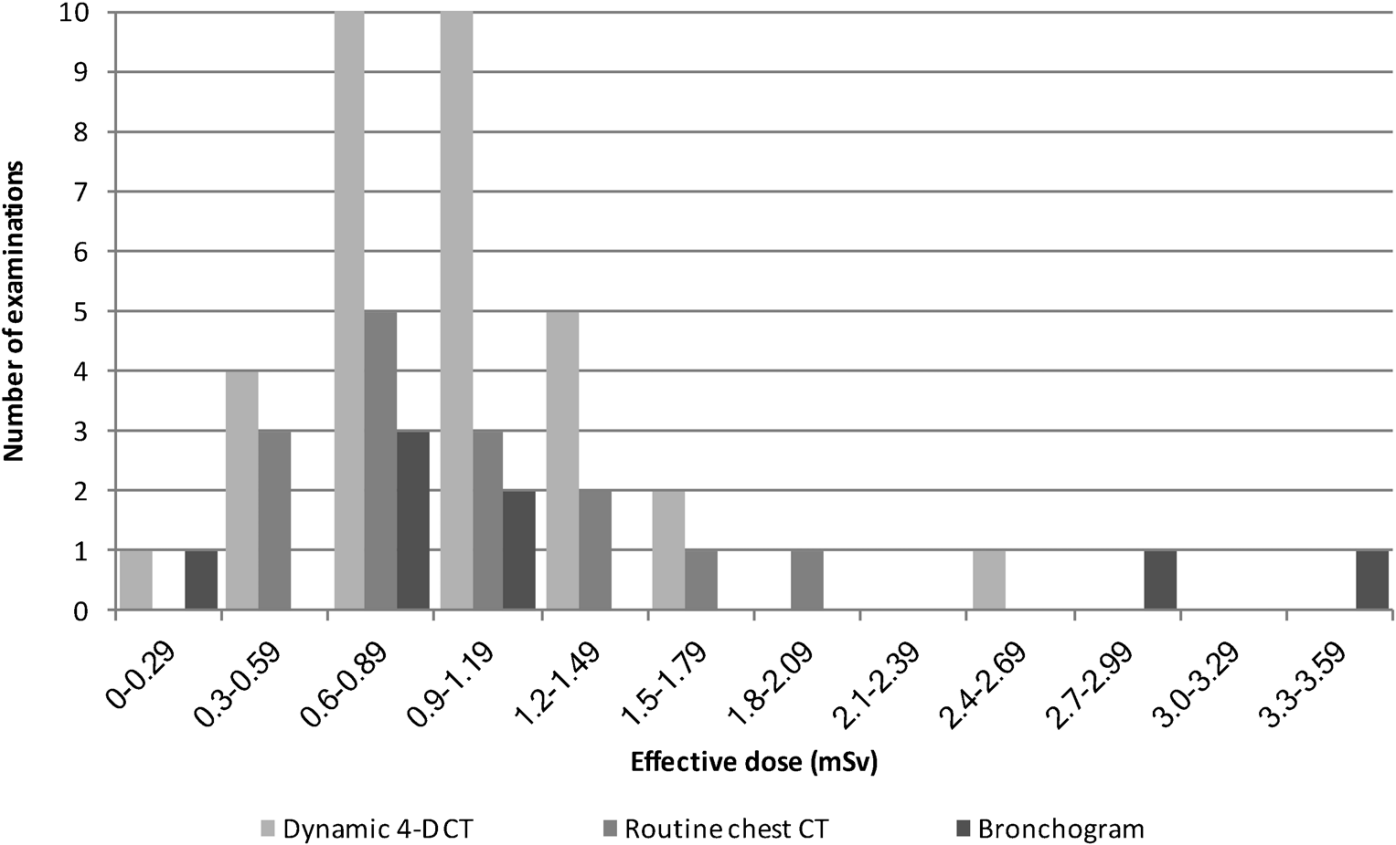
Histogram shows distribution of effective doses for the different examinations

Mean effective dose for a scan was 1.0 mSv and is compared against routine CT chest and bronchograms in Table 2 and Fig. 6.

#### Diagnoses

Of the 33 children, 23 (70%) showed 31 airway stenoses:

10 (32%) tracheal (2 lower; 2 upper, 5 mid and 1 complete), 7 (23%) right bronchial (3 right main; 3 bronchus intermedius, 1 upper lobe bronchus) and 14 (45%) left bronchial (all left main).

According to our classical definition, 12 patients had tracheobronchomalacia (Supplementary material 3). When applying the additional criterion of judging collapsibility in an already stenosed bronchus against normal-appearing proximal airway, eight more patients were diagnosed with tracheobronchomalacia. In the 20 patients (61%) with tracheobronchomalacia, 27 sites were affected -- 8 tracheal, 7 right bronchial and 12 left bronchial. In addition, two tracheal anomalies (6%) were also demonstrated: tracheal bronchus and complete tracheal rings.

**Fig. 7:**
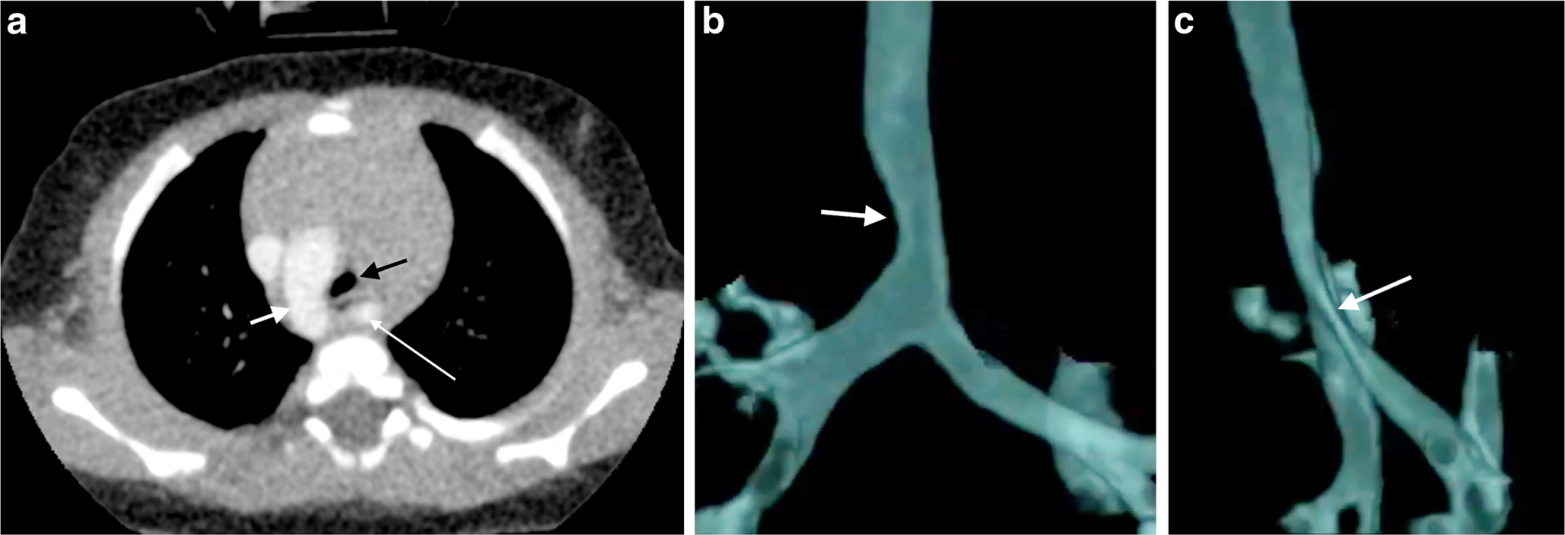
Dynamic 4-D CT study of the airways in a 4-month-old boy with a right aortic arch and aberrant left subclavian artery (the dynamic representation of this imaging is Supplementary material 5). **a** Axial slice of one of the four post-contrast CT scan phases of the dynamic 4-D CT scan demonstrates the right aortic arch (*short white arrow*) and course of the aberrant left subclavian artery (*long white arrow*) in the traditional axial plane. The tracheal compression (*black arrow*) from the right anteriorly by the aorta and posteriorly by the aberrant left subclavian artery is demonstrated. **b** Anterior perspective of a 3-D volume-rendered reconstruction from a dynamic 4-D CT demonstrates a fixed impression on the right side of the trachea (due to the right-side aortic arch) (*white arrow*) bronchial sites of stenosis. **c** Lateral perspective of a 3-D volume-rendered reconstruction from a dynamic 4-D CT demonstrates the severity of the tracheal stenosis best, where the trachea and left main bronchus are compressed from posterior by the aberrant left subclavian artery (*white arrow*)


Cardiovascular abnormalities were demonstrated in 12 patients (36%) using dynamic 4-D CT (Fig. 7 and Supplementary material 5): 3 right aortic arch, 2 aberrant right subclavian arteries, 2 complex cardiac anomalies, 1 cardiomegaly, 1 double aortic arch, 1 pulmonary atresia, 1 bilateral superior vena cava and 1 hypoplastic aortic arch. Of these, nine patients had tracheobronchomalacia. Only eight had been referred with underlying cardiovascular predisposition (Fig. 1).

Supplementary material 5a- c: The 3-D volume-rendered reconstruction of a dynamic 4-D CT scan of the chest in a 4-month-old boy with a right side aortic arch and an aberrant left subclavian artery. (a) View from anterior of the transparent airway reconstruction demonstrates an impression of the right aortic arch on the distal trachea and dynamic reduction of the left main bronchus >28% on free breathing in expiration, in keeping with bronchomalacia. (MP4 1,248 kb)


#### Outcomes (Fig. 8)

Fourteen of 20 children (70%) with tracheobronchomalacia were managed successfully by optimising conservative management: 5 (25%) underwent surgical interventions and 1 (5%) died from the presenting disorder.

**Fig. 8:**
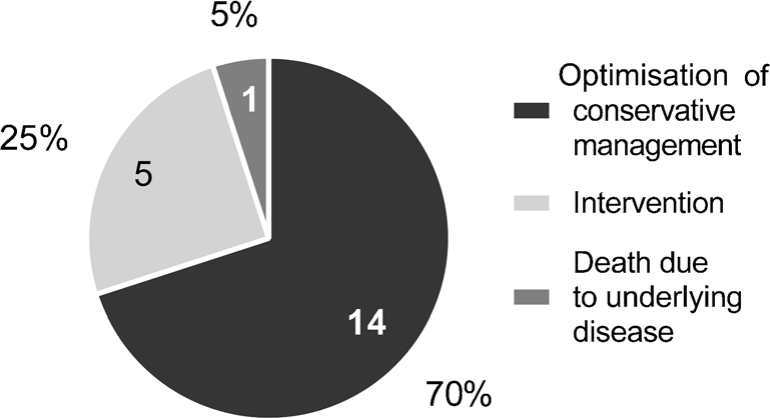
A pie chart of the 20 patients diagnosed with tracheobronchomalacia on dynamic 4-D CT according to the number and proportions in each care/outcome category

## Discussion

Tracheobronchomalacia is the excessive collapse of the trachea and/or bronchi during expiration [5, 11]. Congenital tracheomalacia is the most common congenital tracheal abnormality while acquired tracheomalacia results from an insult, e.g., trauma, external compression, positive pressure ventilation, infection or inflammation [6]. Focal tracheomalacia in children is seen with congenital compression of the trachea or through prolonged intubation and in these patients the trachea is often stenosed on inspiration and collapses further on expiration [3]. Severe tracheobronchomalacia requires treatment [4] dependent on the site, cause and comorbidities [4]. Treatment options include conservative management such as artificial ventilation with high post-expiratory pressure and interventions including aortopexy, tracheostomy, splinting and stent placement [4, 12]. The leading cause of tracheobronchomalacia is vascular compression (48%) [4] and, therefore, aortopexy is frequently used [12]. This technique is also utilised with oesophageal atresia and idiopathic tracheomalacia [4]. Intraluminal stents are less desirable because they can become dislodged or obstructed [12]. Treatment depends on imaging for demonstrating a stenosis, diagnosing tracheobronchomalacia, distinguishing focal tracheobronchomalacia from diffuse tracheobronchomalacia and identifying vascular anomalies.

**Fig. 9:**
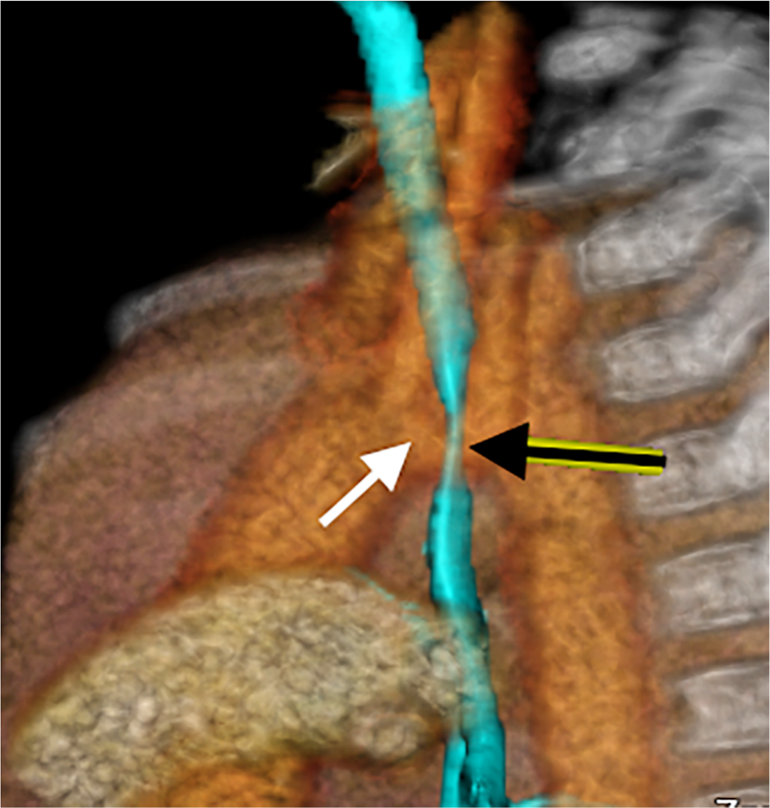
A lateral view of a volume-rendered 3-D reformat created from one phase of a dynamic 4-D CT in an 8-month-old boy with right-side arch and innominate impression on the trachea. The relationship of the vascular structures is demonstrated in orange and yellow, with the airways in transparent light blue. Note the close relationship of the left-positioned brachiocephalic trunk (*white arrow*) and the anterior trachea (*black arrow*), which on cine mode (Supplementary material 6) demonstrates innominate artery compression syndrome


Traditionally, diagnosis of tracheobronchomalacia is by bronchoscopy, which has significant disadvantages: it is invasive [7]; requires general anaesthesia and positive-pressure ventilation (masking tracheobronchomalacia) [5, 13]; it risks complications (including death) [5]; is operator dependent; is subjective, lacking airway measurements (and underestimating collapse), and small airways in infants are not well seen [5, 7, 13]. Fluoroscopy, tracheobronchography, multi-detector CT and dynamic magnetic resonance scanning are also used for diagnosis [6]. Fluoroscopy deserves attention because it is noninvasive, quick, does not require contrast or patient cooperation and yields dynamic information with high specificity (reported to be 94–100%) [13], but it lacks sensitivity (23.8%–62%) [1, 6], is poor at demonstrating anatomical detail, is two-dimensional, is subjective and also underestimates collapse [3, 13]. Contrast bronchography is preferred by many because it shows tracheobronchomalacia can be repeated at varying pressures of ventilation [8, 14] and is more sensitive than bronchoscopy. However, the airway can only be assessed in one plane at a time with overlap of bronchi on the lateral view, again resulting in underestimation of tracheobronchomalacia [13], it is invasive, requires anaesthetic support with intubation and involves injection of contrast material into the airways, risking desaturation from alveolar flooding [13]. Despite these risks, bronchography is considered safe in experienced units [14]. Radiation effective dose from bronchography ranges between 0.26 and 2.47 mSv [13], which is similar to our bronchographic studies (0.3–3.5 mSv). Spirometer-controlled cine magnetic resonance imaging (MRI) is not widely used because it can take from 9 to 20 min to perform, requires patient cooperation and lacks the spatial resolution of CT; thus, it is only feasible for children older than 8 years [5]. CT is a noninvasive alternative, but concerns regarding radiation [5] from imaging during inspiration and expiration (double the dose of radiation) have hampered its widespread use in children [6, 15, 16]. Dynamic 4-D CT has many advantages because it is noninvasive, dynamic (providing airway inspiratory and expiratory information during physiological breathing) [17], fast; high-quality [2, 15]; objective for measurement of airway collapse [3] and craniocaudal extent (i.e. distinguishes focal from diffuse tracheobronchomalacia) [11, 18], demonstrates adjacent structures [3] and it allows 3-D reconstructions [11] (Fig. 9). CT is most useful for simultaneously demonstrating tracheobronchomalacia and any cardiovascular cause [3].

(b)View from the left of the transparent airway reconstruction shows dynamic reduction of the already narrowed left main bronchus of >28% with free breathing in expiration, in keeping with bronchomalacia. (MP4 735 kb)


Short z-axis coverage of narrow-array CT scanners requires helical scanning, resulting in different airway segments being imaged at varying phases of respiration [18], because expiration occurs earlier near the carina than in the more proximal trachea [1, 6]. In contrast, volume scanning allows the entire length of the airway to be scanned simultaneously [1]. Wide-detector scanners such as the 320-detector row CT volumetric scanner used by us, Kroft et al. [19] and Greenberg and Dyamenahalli [7] provide coverage up to 16 cm allowing inclusion of the whole paediatric chest without moving the table [1] [19]. The more modern version of this scanner (Aquilion ONE Vision edition, Otawara, Japan) can acquire at 0.275 s per full rotation if at maximum speed providing isometric, isophasic and isovolumetric 4-D imaging in a real-time respiratory cycle [2], i.e. in non-sedated and non-intubated children [2, 7]. The acquisition includes multiple gantry rotations, divided into separate temporal dynamics (usually 4–5 gantry rotations). Half-scan reconstruction usually allows for the creation of eight dynamic phases from the four rotations, for viewing dynamic airway changes in cine mode [2, 7] as 3-D and multiplanar reconstructions [1]. Reconstructions improve on axial scans when findings are subtle, when determining craniocaudal extent of focal tracheobronchomalacia, for viewing oblique and complex airway anatomy and when communicating information [2] (Supplementary material 6).

Greenberg (in 2012) [1] performed dynamic 4-D CT in 24 infants and small children including intubated patients, much like we did. We based our definition of tracheobronchomalacia of greater than 28% reduction in airway area during expiration when not using forced respiration on previous reports [1, 7]. In 2014, Greenberg and Dyamenahalli [7] reported dynamic 4-D CT in 17 children: 2 of 12 with tracheomalacia had vascular compression and 4 of 8 with left bronchomalacia had atrial or vascular compression. In their patients, dynamic 4-D CT allowed detailed simultaneous evaluation of airway and vascular abnormalities resulting in management changes in 70% [7]. We also demonstrated cardiovascular abnormalities with contrast-enhanced dynamic 4-D CT in 12 patients (36%); of these, 9 had tracheobronchomalacia. Our findings changed management significantly in 5 children (25%) who underwent surgical interventions. Long-term follow-up of these cases is not yet available. In another 14 children (70%), conservative management, including optimisation of ventilation in intensive care patients or watchful waiting in ambulant patients, resulted in good short-term outcomes. One patient died from their underlying cardiac condition.

The pitfalls and poor quality dynamic 4-D CT encountered at the start of our program detract from the success of later studies. At first, we failed to recognize the value of routine intravenous contrast administration in demonstrating unsuspected vascular anomalies, causing secondary tracheobronchomalacia (reported in 20%-81%) [4, 13]; we now administer intravenous contrast routinely. Lee et al. [15] showed 53% prevalence of tracheobronchomalacia in symptomatic children with mediastinal aortic vascular anomalies -- the relevance being that respiratory symptoms from tracheobronchomalacia may persist after surgical correction of the vascular anomaly. The presence of an endotracheal tube in the group referred from neonatal intensive care is also an avoidable pitfall, which can distort the trachea, affect dynamic changes [6] and result in major artefact (Fig. 4). We overcame this pitfall by repositioning the endotracheal tube tip just above the thoracic inlet, using the initial scout view as a guide at the start [11, 20] (Fig. 2). A third avoidable pitfall is an in situ nasogastric tube (18% of our patients) (Fig. 3) (Supplementary material 4), which causes artefact and which we now routinely remove before dynamic 4-D CT. The unavoidable presence of oesophageal air is a prominent feature in crying babies and affects volume-rendered 3-D reconstructions in awake children (Figs. 2 and 5).

Motion artefact, exaggerated by indwelling tubes, affected quality. Even though volumetric CT is up to 24 times faster than helical CT [19], continuous scanning over 1.4 s allowed for motion artefact in 55% of our patients, yet rendered only 1/3 of studies nondiagnostic. The narrative is important again, in that at the start of the service, care of the child during scanning was left to the clinical team and was without anaesthesia in 82% of cases. Anaesthesia is undesirable because it minimizes changes in transmural pressure masking tracheomalacia [6] and is dependent on anaesthetic services. Even with the use of anaesthesia, Lee et al. [17] encountered motion artefact in 20% of their combined inspiratory and expiratory dynamic CT in children. Some movement artefact is unavoidable because the position of the tracheobronchial tree changes during the respiratory cycle in the craniocaudal direction. Dynamic 4-D CT is not affected by the craniocaudal movement of the airway [13], but anteroposterior movement can cause the airway to be out of plane for minimum intensity coronal slabs. Presence of a gowned radiologist within the scanner room until immediately preceding the scan and use of a vacuum immobilisation device (RedVac VMR433X01, VMR438X01; Kohlbrat and Bunz, Austria) improved our quality. We also improved quality by reconstructing a half rotation of projection data rather than a full one, improving temporal resolution and creating smoother cine-loops.

Effective radiation doses from paired inspiratory and expiratory multi-detector CT range between 3.5 and 7.5 mSv [11], but recent studies have shown reductions of up to 23% [6, 13, 16]. Low-dose studies are diagnostic, despite increased image noise due to the natural high contrast of the airway and lungs [1, 11, 13, 16]. Volume CT imparts up to 40% lower radiation doses because there is none of the *z*-overranging associated with helical CT and less overbeaming (penumbra effect) [1, 19]. We also recommend limiting the craniocaudal acquisition from just below the vocal cords to 3 cm below the level of the carina [11, 13, 19] except where whole-lung demonstration is needed (Supplementary material 7); limiting continuous scan time to a single breath [1], using iterative reconstruction [7] and switching to an Adaptive Iterative Dose Reduction reconstruction algorithm [7] for further radiation dose reduction. Greenberg [1] initially (2012) reported a mean effective dose of dynamic 4-D CT in children of 1.7 mSv (standard deviation [SD], 1.1 mSv), which improved to 1.1 mSv (range: 0.4–1.9 mSv) in 2014 [7]. This matches our experience and compares favourably against bronchography [13] but contrasts with a 2005 report by Mok et al. [14] where bronchography (0.27–2.47 mSv) performed better than helical CT (0.86–10.67 mSv). Our benchmarking against bronchogram doses (mean: 1.4 mSv; range: 0.3–3.5 mSv) compares well with the report by Mok et al. [14] but exceeds our dynamic 4-D CT doses. Volume scan mode is only available with specific vendors, but no additional kit is required and there is no extra cost once the scanner is purchased. Our scanner was installed for imaging major trauma and the possibilities from volume scanning were only recognised later. Therefore, no additional funding was required. Since the study is performed with free breathing, no anaesthetic support is required for setting up and continuing the service. Apart from radiographer and radiologist buy-in, referral departments need to learn about the new technique, its advantages, limitations and possible indications. Our experience of a gentle start performing dynamic 4-D CT only for patients in whom one of the traditional techniques is either not possible or has failed allows the team to perfect technical aspects and develop skill. The impressive visuals from dynamic 4-D CT sell themselves. Standard of care changes when referrals for the new technique push old techniques into obsoleteness.


(c)View from posterior of the transparent airway reconstruction, with superimposed vascular structures in light brown, demonstrates an impression of the right aortic arch on the distal trachea and the aberrant left subclavian artery coursing diagonally over the left main bronchus. (MP4 4,850 kb)


As acknowledged in previous publications [1, 7], not all patients had correlative studies such as bronchoscopy, which is a limitation of this study, but the findings are accepted because of the established accuracy of CT.

## Conclusion

We recommend dynamic 4-D CT as an achievable, low-dose, one-stop-shop imaging technique for diagnosing tracheobronchomalacia and its vascular causes through several referral patterns because it impacts management decisions for surgery or for optimising conservative management. The routine use of intravenous contrast, removal of nasogastric tubes, withdrawal of indwelling endotracheal tubes into the upper trachea and use of vacuum restraining devices will help novice users avoid many of the pitfalls and improve quality.

## Electronic supplementary material

Supplementary material 6 The 3-D volume-rendered reconstructions of a dynamic 4-D CT scan of the airways in an 8-month-old boy, with innominate artery compression of the trachea (not recorded as pathologic in our study). (a) View from the right, with the airways in transparent blue and the vessels in light brown, demonstrates the brachiocephalic trunk impressing and deforming the anterior trachea but there are no features of tracheomalacia. (MP4 2,806 kb)

(b) View from the posterior, with the airways in transparent blue and the vessels in light brown, demonstrates the brachiocephalic trunk to the left of midline and directly in contact with the anterior trachea but without any features of tracheomalacia. (MP4 3,350 kb)

Supplementary material 7 The 3-D volume-rendered reconstruction of a dynamic 4-D CT scan of the airways and whole lungs in a 3-month-old boy viewed from the anterior. There is bronchomalacia involving the right upper lobe bronchus, the right lower lobe bronchus and the left main bronchus. The inclusion of the lungs in this patient demonstrates the dynamic segmental air involving the right upper lobe anterior segment and the lateral segment of the middle lobe compared to the static air-trapping affecting the whole left upper lobe. This results in paradoxical movement of the apices of the lungs in comparison to the coordinated movement of the diaphragm. (MP4 5,544 kb)
